# Inference of drowning sites using bacterial composition and random forest algorithm

**DOI:** 10.3389/fmicb.2023.1213271

**Published:** 2023-06-27

**Authors:** Qin Su, Chengliang Yang, Ling Chen, Yiqing She, Quyi Xu, Jian Zhao, Chao Liu, Hongyu Sun

**Affiliations:** ^1^Faculty of Forensic Medicine, Zhongshan School of Medicine, Sun Yat-sen University, Guangzhou, China; ^2^Guangzhou Forensic Science Institute, Guangzhou, China; ^3^School of Forensic Medicine, Southern Medical University, Guangzhou, China; ^4^Guangzhou Municipal Public Security Bureau, Guangzhou, China; ^5^National Anti-Drug Laboratory Guangdong Regional Center, Guangzhou, China; ^6^Guangdong Province Translational Forensic Medicine Engineering Technology Research Center, Sun Yat-sen University, Guangzhou, China

**Keywords:** drowning site, 16S rDNA amplicon sequencing, random forest, Pearl River, bacterial composition

## Abstract

Diagnosing the drowning site is a major challenge in forensic practice, particularly when corpses are recovered from flowing rivers. Recently, forensic experts have focused on aquatic microorganisms, including bacteria, which can enter the bloodstream during drowning and may proliferate in corpses. The emergence of 16S ribosomal RNA gene (16S rDNA) amplicon sequencing has provided a new method for analyzing bacterial composition and has facilitated the development of forensic microbiology. We propose that 16S rDNA amplicon sequencing could be a useful tool for inferring drowning sites. Our study found significant differences in bacterial composition in different regions of the Guangzhou section of the Pearl River, which led to differences in bacteria of drowned rabbit lungs at different drowning sites. Using the genus level of bacteria in the lung tissue of drowned rabbits, we constructed a random forest model that accurately predicted the drowning site in a test set with 100% accuracy. Furthermore, we discovered that bacterial species endemic to the water were not always present in the corresponding drowned lung tissue. Our findings demonstrate the potential of a random forest model based on bacterial genus and composition in drowned lung tissues for inferring drowning sites.

## 1. Introduction

Drowning is a significant global cause of unnatural death (World Health Organization, 2014). However, the body discovery site in water is often not the actual drowning site (Saini et al., 2017). Determining the precise drowning site is crucial in forensic investigations (Fang et al., 2019). During the drowning process, small objects such as planktonic microorganisms present in the water can be aspirated into the lungs along with water and then breach the pulmonary blood barrier and circulate through the aorta, reaching various major organs such as the liver, kidney, and bone marrow (Lee et al., 2017). Therefore, inferring the drowning site primarily involves analyzing whether the drowned victim’s organs contain the same specific markers as those found in the suspected drowning water (Siver et al., 1994; Coelho et al., 2016). These markers, used to infer the drowning site, are typically widespread and exhibit a specific distribution in drowning waters.

Previous research has primarily focused on identifying markers such as foreign bodies, diatoms, and other plankton (He et al., 2008; Zhang et al., 2020; Seena et al., 2022). Foreign bodies are exogenous substances that are naturally present in water but typically absent or found in small amounts in the human body, such as small particle colonies containing silicon, aluminum, or calcium. Foreign bodies are usually specific to certain types of water, including sewage outlets of chemical plants, steel plants, and military factories. However, this method’s applicability is limited in cases where industrial discharge is unstable or absent. Diatoms are unicellular photoautotrophic algae widely distributed in water bodies, with an estimated species diversity of up to 100,000 (Mann and Vanormelingen, 2013). The composition of diatom species in water is significantly influenced by the environment, exhibiting distinct regional characteristics (Thakar and Singh, 2010; Carballeira et al., 2018). Many researchers have devoted larger efforts to determining the drowning site by comparing the diatom groups between suspicious water samples and tissues of drowned corpses (Digerfeldt, 1987; Ludes et al., 1999; Horton et al., 2006). However, current diatom testing methods for forensic pathologists are laborious, time-consuming, and often require specialized knowledge. Additionally, the application of deep learning technology used for automatic diatom identification is still in its early stages, and practical implementation is not yet feasible (Zhang et al., 2021). Furthermore, when the water lacks specific diatoms or the large volume of diatoms restricts their entry into the circulation of the drowned corpse, diatom tests play no role in inferring the drowning site (Alan and Sarah, 2012). Another potential marker is the ribulose-1,5-bisphosphate carboxylase large-chain gene (*rbcL*) in phytoplankton (Fang et al., 2019). However, the usefulness of *rbcL* is limited by the presence of nonspecific products in the water sample that can interfere with the results.

Aquatic bacterial populations exhibit significant variations across different habitats, such as seawater, freshwater, and sewage, and they are much smaller in size (0.2 ~ 2 μm) than diatoms (2 to >500 μm) (Uchiyama et al., 2012). Additionally, bacteria outnumber diatoms by a large margin (Armbrust, 2009). Consequently, it is easier to retrieve aquatic bacteria from drowned corpses compared to diatoms (He et al., 2008). Researchers such as Kakizaki et al. have proposed the use of pyrosequencing microbiome analysis to examine drowned corpses and have reported the presence of an aquatic microbiome in the blood and closed organs of drowned individuals (Kakizaki et al., 2012). With advancements in second-generation sequencing technology and improvements in bioinformatics (Bolyen et al., 2019), 16S rDNA amplicon sequencing has become a widely employed tool in forensic research. Its applications include drowning diagnosis (Wang et al., 2020), forensic soil analysis (Jesmok et al., 2016), and postmortem time inference (Dong et al., 2019).

However, to the best of our knowledge, there have been no previous reports on the utilization of aquatic bacteria for inferring drowning sites. To explore the feasibility of using aquatic bacteria for this purpose, we conducted a 16S rDNA amplicon sequencing analysis. The aim was to determine whether the drowned corpse exhibits the same aquatic bacterial composition and endemic aquatic bacteria as those found in the suspected drowning water area.

## 2. Materials and methods

### 2.1. Study sites and water samples

**Figure 1:**
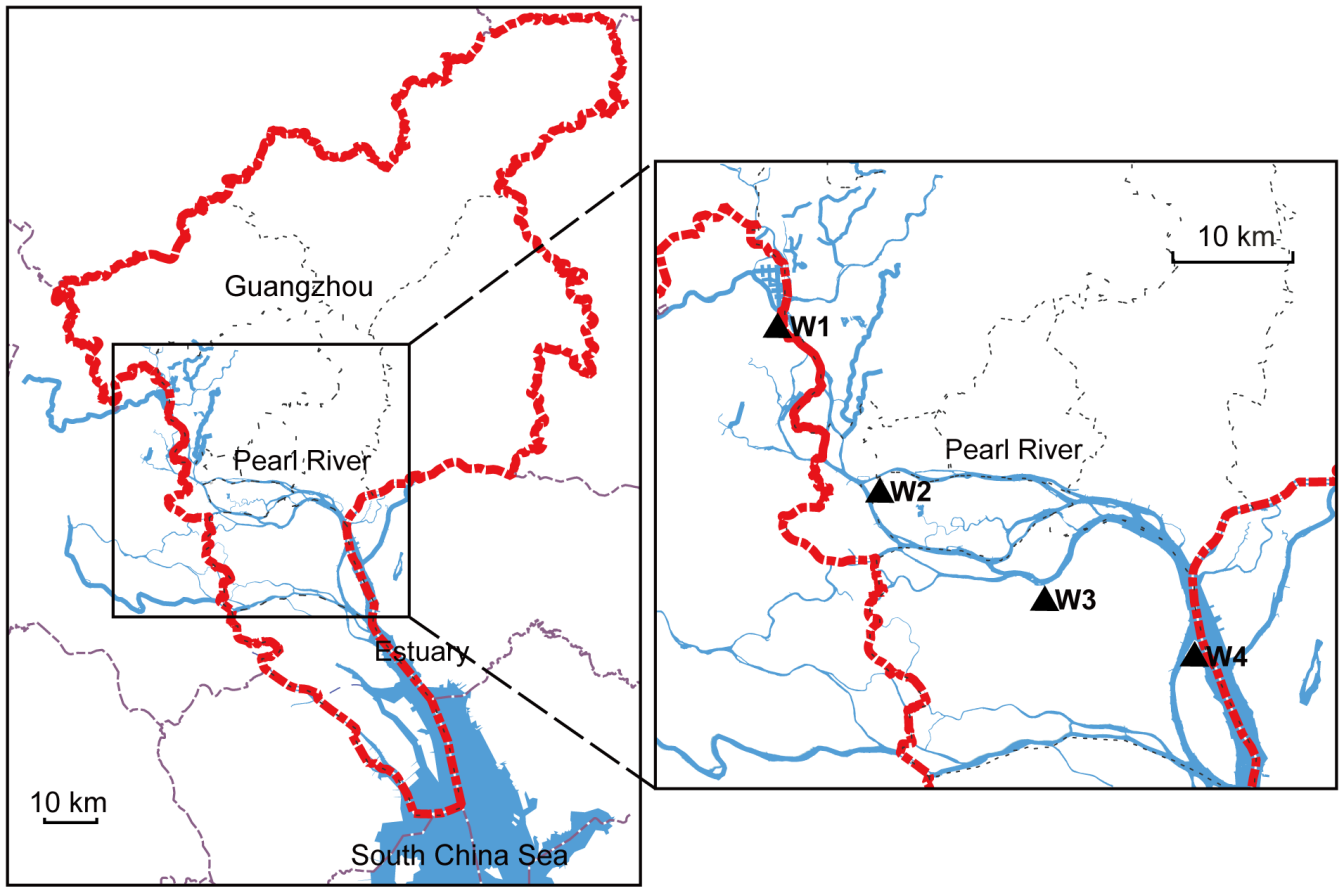
Map of study sites in the Pearl River. The left side shows the Guangzhou area indicated by the red dotted line. The blue line represents the Pearl River, and the right side highlights the sampling locations using black triangles.

To investigate the distribution of aquatic bacteria in the Guangzhou section of the Pearl River, we selected four random sampling locations (namely, W1–W4), as depicted in Figure 1. At each designated location, we employed autoclaved plastic bottles to collect a total of five water samples. Each sample, standardized at a volume of 15 mL, was carefully extracted from a depth exceeding 30 cm beneath the river’s surface. The sampling process was conducted within a well-defined and contiguous area measuring 100 cm × 100 cm, positioned at a distance of 150 cm away from the riverbank. Immediately after collection, the water samples were transported to the forensic laboratory. To enrich the aquatic bacteria, we filtered the samples using the HL-6 multiunit vacuum suction filter. The filter membranes used had a pore size of 0.22 μm. After filtration, the filter membranes were carefully placed in 50 mL sterile centrifuge tubes for further analysis. To preserve the samples, all collected samples were rapidly frozen in liquid nitrogen and stored at −80°C. Additionally, 50 L of water was collected at each sampling location within the same defined area for subsequent animal experiments, and all water samples were used within 24 h.

### 2.2. Animal experiments and tissue samples

Female New Zealand rabbits, aged 3 months and weighing between 1.8 and 2.2 kg, were obtained from the Laboratory Animal Center of Southern Medical University (n = 24). At each sampling location, six rabbits were submerged in the respective water samples until they died. The drowned rabbits were then kept in separate water samples for 24 h before their lungs were removed. The drowned rabbit lungs from different sampling locations were labeled DL1, DL2, DL3, and DL4. After removal, the rabbit lungs were promptly frozen in liquid nitrogen and stored at −80°C. All animal experiments were conducted in accordance with the guidelines and regulations approved by the Animal Ethics Committee of Southern Medical University (L2020064).

### 2.3. DNA extraction

To isolate DNA from the filtered water samples, filter membranes were cut using sterilized scissors and mixed with 20 μL of proteinase K and 20 μL of DTT. The DNeasy PowerSoil Kit (Qiagen, Hilden, Germany) was employed for DNA extraction. Tissue samples were homogenized in a mortar with liquid nitrogen, and DNA extraction from tissues was performed using the E.Z.N.A. Water DNA Kit (Omega, USA) following the manufacturer’s instructions.

### 2.4. PCR amplification and sequencing

The V3-V4 regions of the 16S rDNA were amplified using primers 340F and 805R (340F, 5′-CCTACGGGNBGCASCAG-3′, 805R, 5′-GACTACNVGGGTATCTAATCC-3′). The first PCR mixture contained 12.5 μL of 2 × KAPA HiFi HotStart Ready Mix Buffer (Kapa Biosystems, Wilmington, MA, USA), 2.5 μL of extracted DNA (5 ng/μL), and 5 μL of each primer (1 μM). The amplification process consisted of an initial denaturation-activation step at 95°C for 3 min, followed by 25 cycles of denaturation at 95°C for 30 s, annealing at 55°C for 30 s, and extension at 72°C for 30 s. The PCR products were confirmed using 2% agarose gel electrophoresis, purified by magnetic beads, and utilized as templates for the second PCR. The second PCR conditions were identical to those of the first PCR. Each amplicon (300 ng) was pooled and further purified using the MoBio UltraClean PCR cleanup kit. Finally, the samples were sequenced on the Illumina NovaSeq 6000 platform with PE250 sequencing. The relevant raw 16S rDNA sequencing data have been deposited in the GenBank repository under accession number PRJNA962514.

### 2.5. Bioinformatic and statistical analyses

The raw data underwent quality control using fastp software (version 0.20.0^1^) (Chen et al., 2018), and sequence merging was performed using FLASH software (version 1.2.7^2^) (Mago and Salzberg, 2011). The resulting sequences were denoised using Deblur (Amir et al., 2017) and imported into QIIME2 (version 138) (Bolyen et al., 2019). The latest SILVA database for QIIME2 was downloaded from the official QIIME2 website at https://docs.qiime2.org/2023.2/. After assigning taxonomy and correcting 16S rDNA copy numbers using classify-sklearn, the data were collapsed at different levels. Pairwise comparisons of the Observed_OTUs and Shannon index were conducted using the Kruskal–Wallis test. Statistically significant (*p* < 0.05) differences between groups are denoted by different lowercase letters. Heatmaps were generated based on the relative abundance of bacteria at the phylum (all) and genus (Top50) levels using the tool available at https://www.omicstudio.cn/tool/4. The Venn diagram was created using the Venn Diagram Plotter tool found at http://bioinformatics.psb.ugent.be/webtools/Venn/. The UpSet plot and random forest model were performed using the R packages UpSetR 1.4.0 and randomForest, respectively. For random forest, the dataset was divided into a training set (67%) and a test set (33%). The key tree-related parameters, such as the number of trees (500) and the tree depth (3), were tuned. The classification accuracy was then used to determine the overall rate of correctly classified samples.

## 3. Results

### 3.1. Location effect of water samples

**Figure 2:**
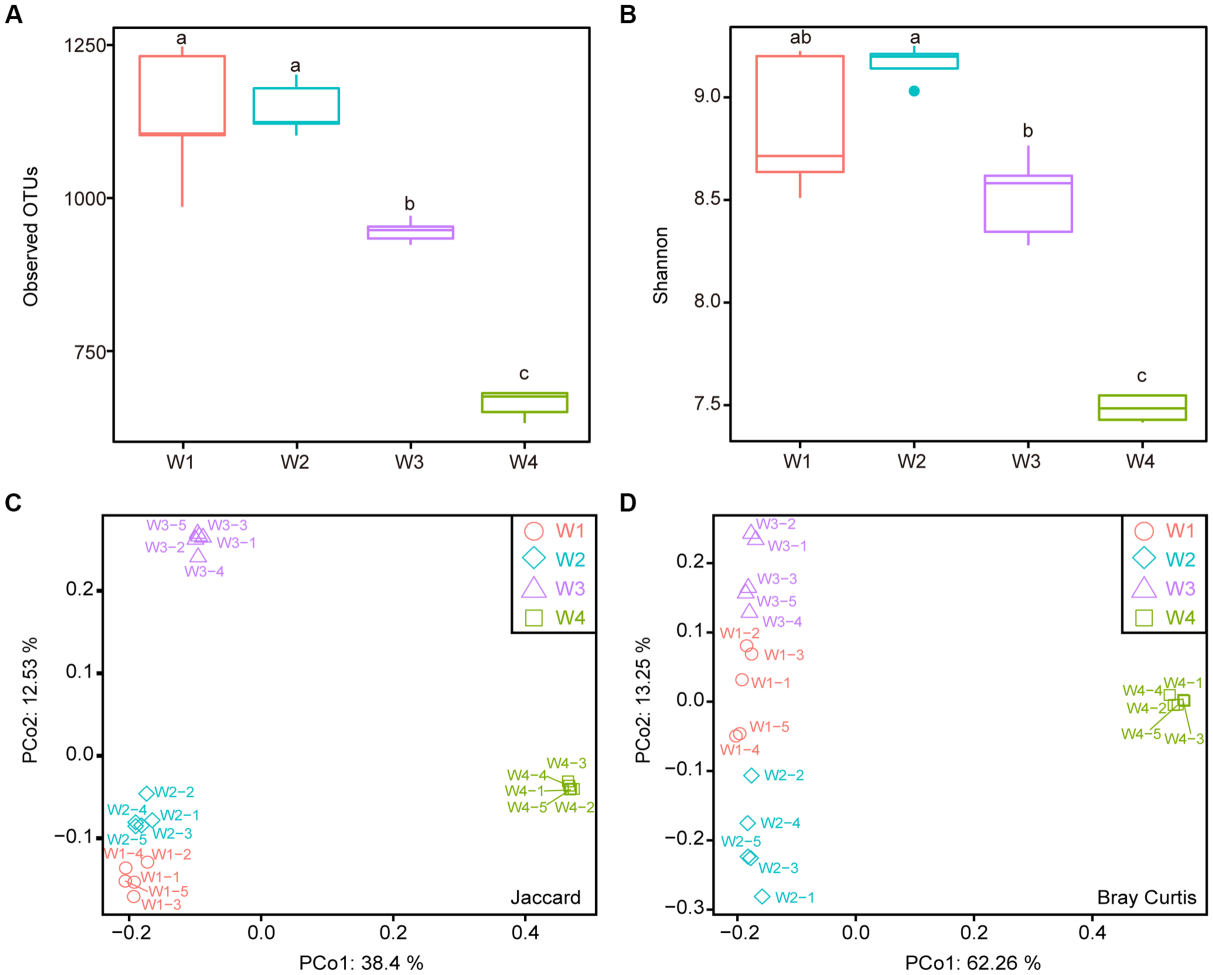
Alpha diversity and beta diversity analysis of four water samples. **(A)** Observed OTUs. **(B)** Shannon index. **(C)** Principal Coordinate Analysis (PCoA) based on Jaccard distance. **(D)** PCoA based on Bray-Curtis distance. Different lowercase letters indicate statistically significant differences between groups (*p* < 0.05, Kruskal-Wallis test).

A total of 4,592 operational taxonomic units (OTUs) were detected in 20 samples. The number of OTUs detected in the water samples decreased with decreasing distance from the estuary, with the highest number observed in W1 (1154.4 ± 35.45) and the lowest in W4 (658.4 ± 7.77) (Figure 2A). The Shannon index, which indicates bacterial diversity, followed a similar trend, with W1 showing the highest diversity and W4 the lowest (Figure 2B). Although there was no significant difference in the observed OTUs and Shannon index between W1 and W2, W1 had more OTUs than W2, while W2 had more diversity than W1, indicating that the species distribution of W2 was more even. To further compare the diversity of these four sampling locations, principal coordinate analysis (PCoA) based on the Jaccard and Bray–Curtis distances was performed. The results showed that W4 was distinct from the other three groups (Figures 2C,D). When only considering the species of bacteria, all four groups could be significantly distinguished, while the species types of W1 and W2 were more similar (Figure 2C). However, when considering the abundance of each species, the differences between groups were relatively reduced, particularly among W1, W2, and W3 (Figure 2D). The analysis of water samples revealed significant effects of location on bacterial diversity and species distribution.

### 3.2. Bacterial community structures of water samples

**Figure 3:**
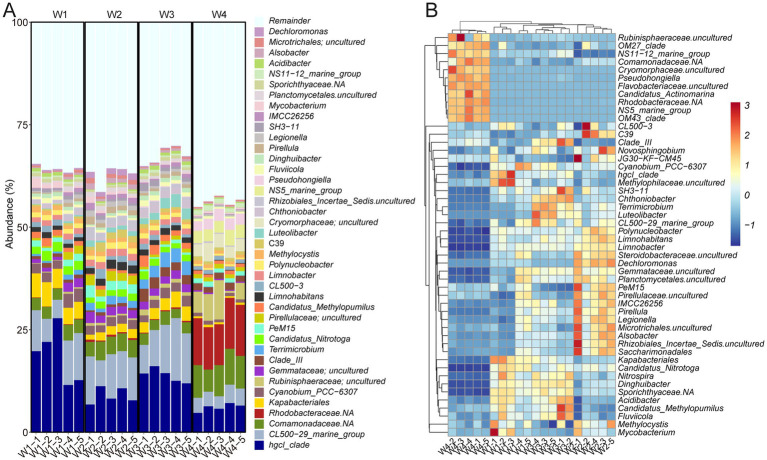
Bacterial taxonomy of water samples at the genus level. **(A)** Bar plot. **(B)** Heatmap displaying the top 50 taxa.

To characterize the bacterial communities in the sampling locations, OTUs were annotated using the Silva 138 database with 99% similarity. At the phylum level, Proteobacteria (38.33% ± 2.32%) was the most abundant phylum in all samples, followed by Actinobacteriota (26.45% ± 1.62%), Bacteroidota (10.91% ± 0.67%), and Planctomycetota (8.07% ± 0.54%) (Supplementary Figure 1A). Notably, Cyanobacteria and Verrucomicrobiota were found to be highly enriched in W1, W2, and W3, while their abundance was lower in W4. Conversely, Thermoplasmatota, Crenarchaeota and Marinimicrobia were more abundant in W4 than in the other three groups (Supplementary Figure 1B). At the genus level, *hgcI_clade* and *CL500-29_marine_group* exhibited the highest prevalence, accounting for 11.89 ± 1.34 and 8.26% ± 0.81%, respectively (Figure 3A). Both *Comamonadaceae* and *Rhodobacteraceae* were prominently represented in W4 but to a lesser extent in the other groups (Figure 3B). Strikingly, *Hgcl:clade* showed the opposite trend. These findings indicate a highly diverse distribution of bacterial composition across the different water sampling locations.

### 3.3. Effects of drowning sites on the bacteria of rabbit lung tissue

**Figure 4:**
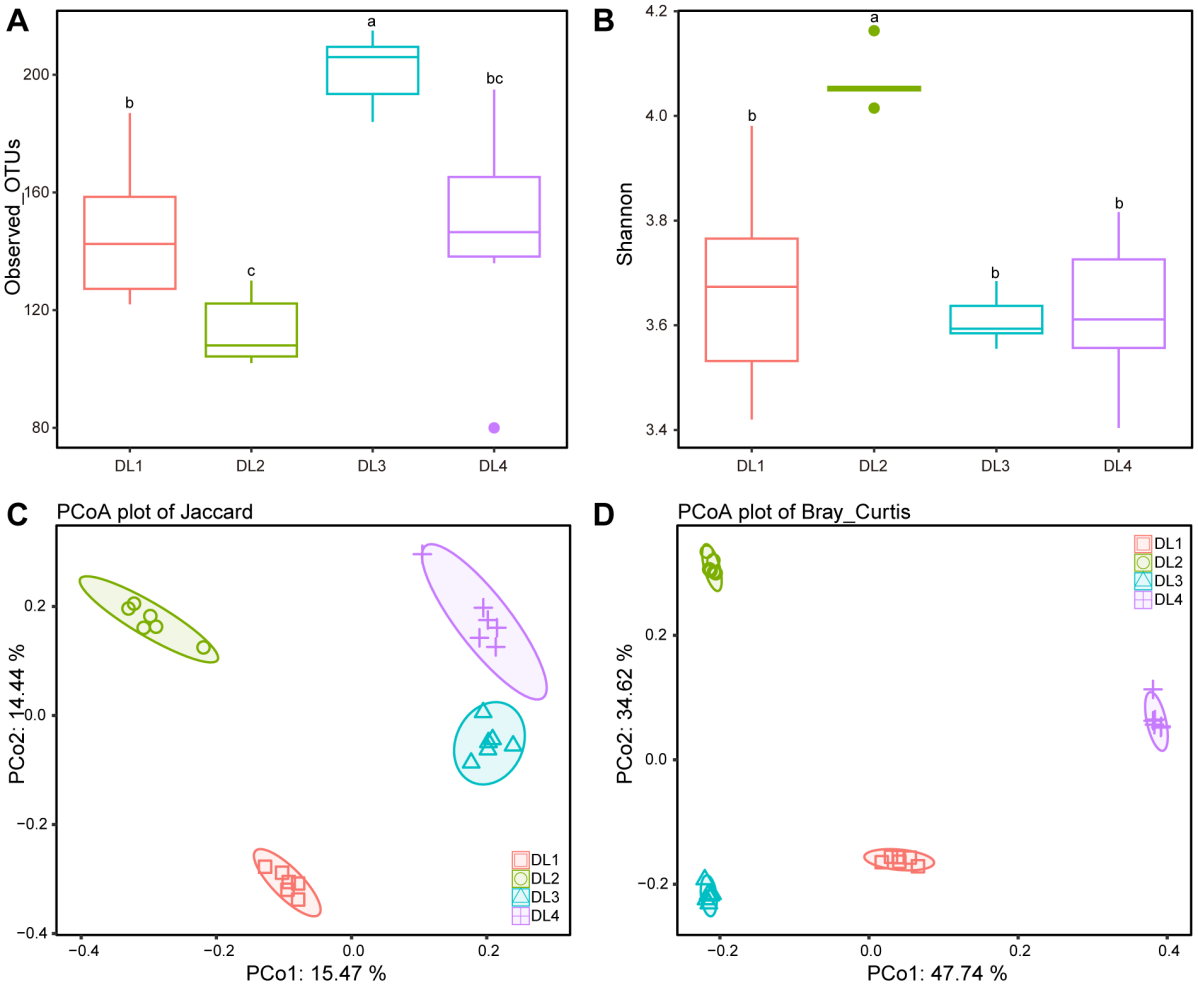
Alpha diversity and beta diversity analysis of four drowned samples. **(A)** Observed OTUs. **(B)** Shannon index. **(C)** PCoA based on Jaccard distance. **(D)** PCoA based on Bray–Curtis distance. Different letters above boxes indicate statistically significant differences between groups (*p* < 0.05).

We examined the bacterial composition of the lung tissue of drowned rabbits at different water sampling locations and observed significant differences in the number of observed OTUs. Among the samples, DL3 exhibited the highest number of OTUs at 208.33 ± 5.71 (Figure 4A). Additionally, the Shannon index was the lowest for DL3 at 3.63 ± 0.04, while it was highest for DL2 at 4.05 ± 0.02 (Figure 4B). Based on Jaccard or Bray–Curtis distance, the PCoA analysis showed that the four sample groups could be independently clustered and distinguished from each other (Figures 4C,D). When the species content (Bray–Curtis distance) was considered, the differences among the four groups were more pronounced (Figure 4D). The dominant families identified in the samples included Aeromonadaceae, Enterobacteriaceae, Fusobacteriaceae, and Peptostreptococcaceae (Supplementary Figure 2). To further investigate the community relationship between the drowned rabbits and water samples, PCoA was performed. PCo1 could differentiate the rabbit lung tissue from the water samples based on both Jaccard and Bray–Curtis distances (Supplementary Figure 3), making it challenging to establish a direct connection between the water samples and the rabbit lung tissue.

### 3.4. Random forest model for inferring the drowning site

**Figure 5:**
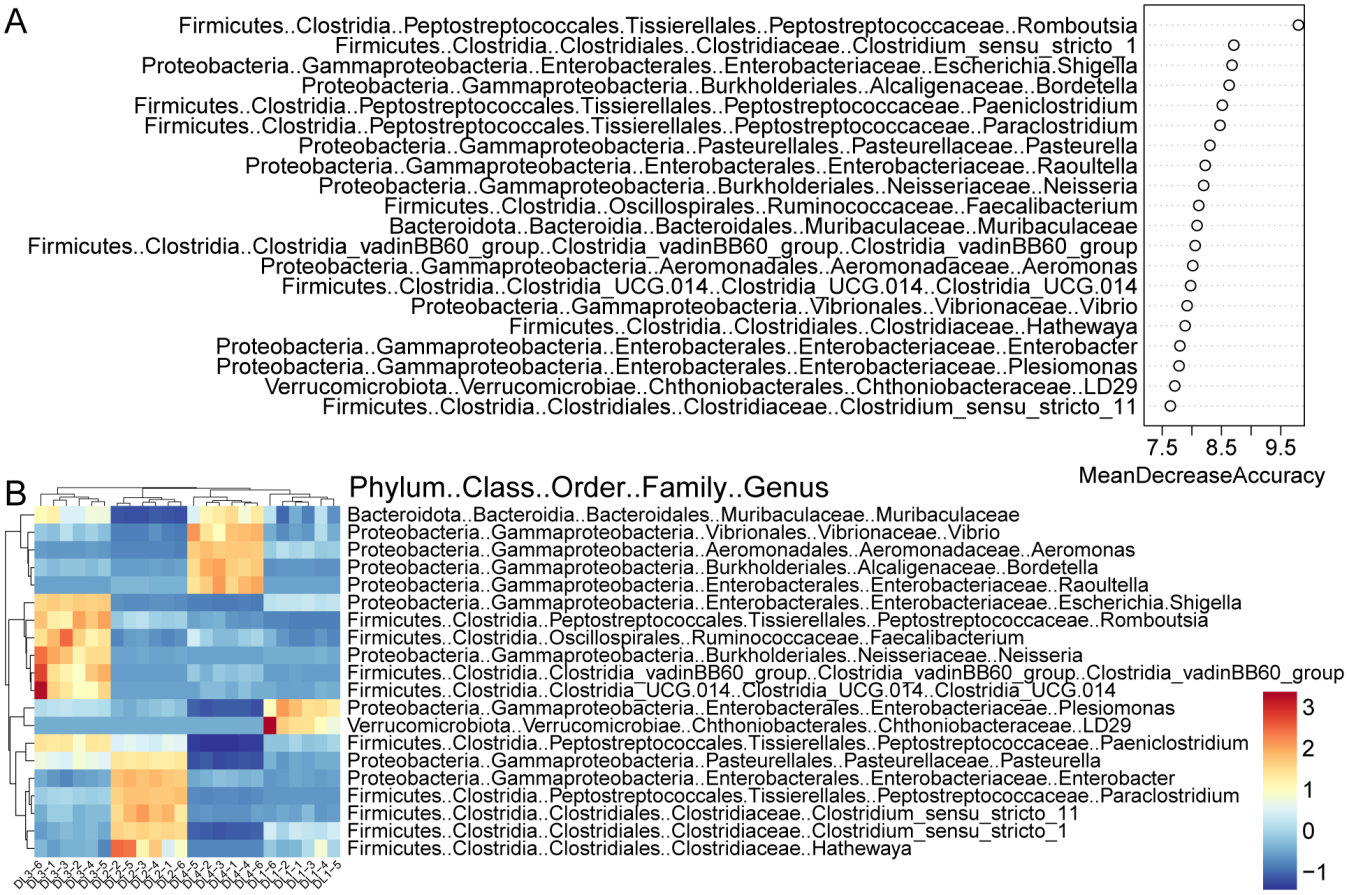
Random forest screening for bacterial taxonomic biomarkers. **(A)** Results of the mean decrease accuracy method in a random forest classifier. The x-axis represents the importance index, and the y-axis represents the bacterial taxonomic biomarkers. The top 20 biomarkers ranked by importance coefficient are displayed. **(B)** Unsupervised clustering heatmap showing hierarchical clustering results of the top 20 bacterial taxonomic biomarkers identified by random forest model.

To manage the large amount of data produced by high-throughput sequencing, the random forest algorithm was utilized to select characteristic biomarker taxa at the genus level. As a feature selection procedure, the mean decrease in accuracy of the top 20 bacterial taxa is presented in Figure 5A. *Rombutsia* was the most significant bacterial taxon in distinguishing drowning sites. Some specific biomarkers of these top 20 bacterial taxa were identified in the lung tissue of drowned rabbits from different drowning sites. As demonstrated in Figure 5B, *LD29* and *Plesiomonas* were enriched in DL1, while *Hathewaya*, *Clostridium_sensu_stricto_1*, *Clostridium_sensu_stricto_11*, *Paraclostridium*, *Enterobacter*, and *Pasterurella* were enriched in DL2. *Clostridia_UCG.014*, *Clostridia_vadinBB60_group*, *Neisseria*, *Faecalibacterium*, *Romboutsia*, *Shigella*, and *Paeniclostridium* were enriched in DL3. *Raoultella, Bordetella*, *Aeromonas*, and *Vibrio* were enriched in DL4. Notably, *Muribaculaceae* was significantly enriched in both DL3 and DL4.

To evaluate the impact of these genus-level characteristic biomarker taxa on predicting drowning sites, a random forest model was built, and the test set was used for prediction. The prediction results are presented in Table 1, showing a prediction accuracy of 100% and a probability of predicting the exact site of ≥65.92%. These results suggest that the random forest model based on 16S rDNA data is highly effective in predicting drowning sites.

**Table 1:** Drowning sites assignment probabilities of the testing samples from four groups of lung samples obtained with a random forest (RF) algorithm classifier based on multiple classification.

SampleID	DL1_pro	DL2_pro	DL3_pro	DL4_pro	prediction	real
DL1-4	0.7298	0.083	0.0842	0.103	DL1	DL1
DL1-5	0.7532	0.0906	0.071	0.0852	DL1	DL1
DL2-1	0.1132	0.7442	0.0544	0.0882	DL2	DL2
DL2-4	0.1216	0.6988	0.0614	0.1182	DL2	DL2
DL3-4	0.117	0.0666	0.6812	0.1352	DL3	DL3
DL3-6	0.0884	0.0886	0.6644	0.1586	DL3	DL3
DL4-2	0.0984	0.0502	0.1052	0.7462	DL4	DL4
DL4-5	0.125	0.0734	0.1424	0.6592	DL4	DL4

The color-coded ones indicate the samples correctly predicted by the random forest model and their probability distributions.

### 3.5. Endemic species for inferring the drowning site

**Figure 6:**
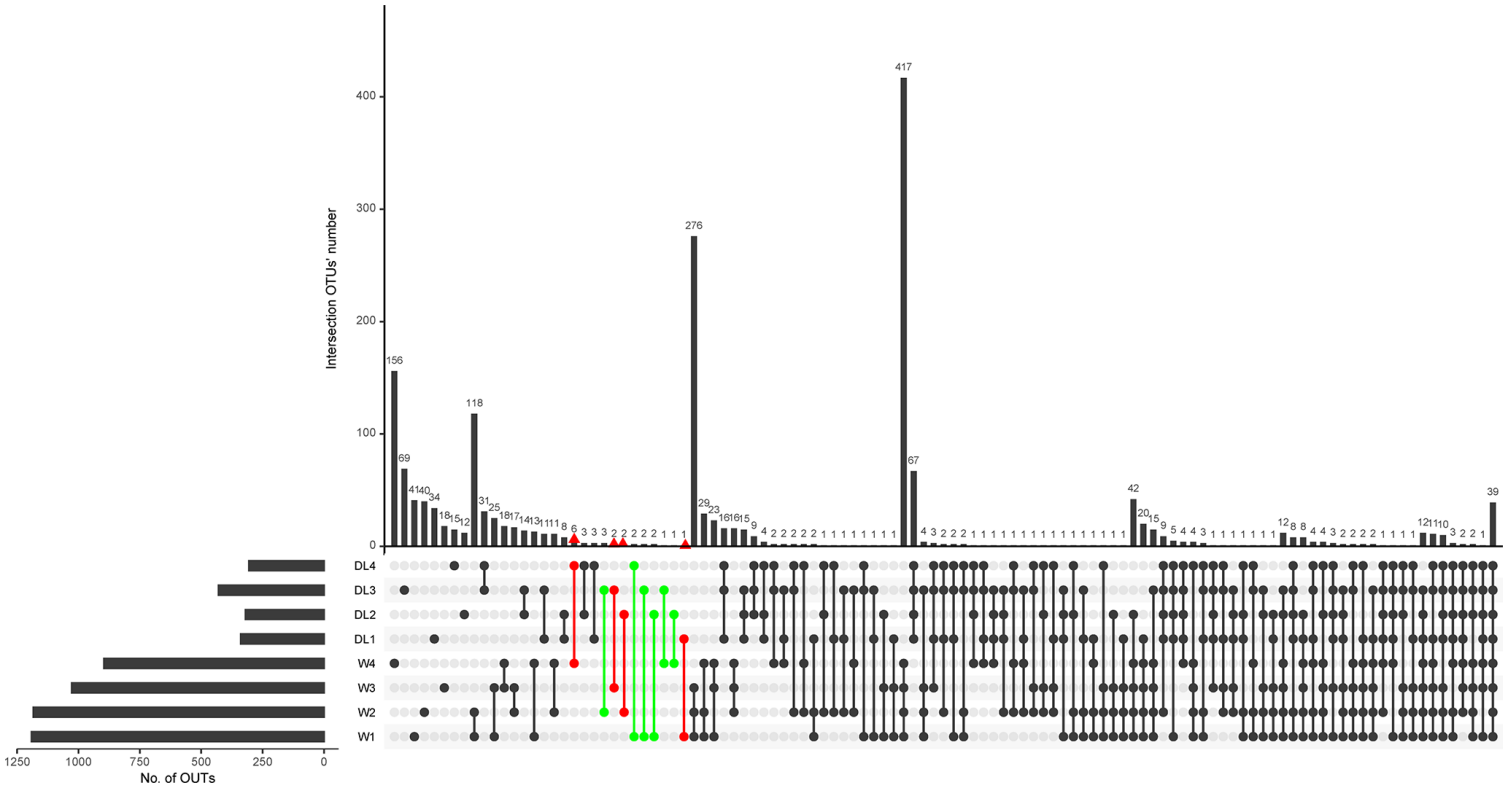
UpSet plot of water samples and drowned samples. The parts of interest are marked with red and green colors.

Among the 4,874 OTUs identified in the water samples, 1,528 were annotated to the species level. Venn analysis revealed that W1, W2, W3, and W4 had 58, 62, 25 and 175 endemic species, respectively (Supplementary Figure 4). To determine if these endemic species were present in the drowned samples, an UpSet plot was generated at the species level. DL1, DL2, DL3, and DL4 had 1, 1, 2, and 6 endemic species, respectively, which were also found in the corresponding water samples (in red, Figure 6). Additionally, some drowned samples contained endemic species from other sampling locations (in green, Figure 6). These findings indicate that the 16S rDNA sequencing method may produce both false positives and false negatives, rendering it unreliable for inferring the drowning site based on endemic species.

## 4. Discussion

The presence of diatoms in organs such as lungs, liver, kidneys, and bone marrow is commonly used as evidence of drowning, and comparing the species and density of diatoms in water samples and organs can help infer the drowning site (Hürlimann et al., 2000; Carballeira et al., 2018; Zhang et al., 2021). However, this approach is challenging to generalize due to the scarcity and dispersal of diatoms throughout the bodies of drowned individuals, as well as the need for specialized expertise in diatom classification. To overcome these challenges, new techniques have been developed, such as using 16S rDNA of microplankton for molecular diagnosis of drowning (Kane et al., 1996) or employing high-throughput sequencing methods such as 454 pyrosequencing to detect aquatic bacteria in blood and organs (Kakizaki et al., 2012). These advancements in microbiome research have heightened the significance of microbiome analysis in forensic applications, enabling rapid identification of microbial species and composition in tissue and water samples (Alan and Sarah, 2012; Clarke et al., 2017).

Studies in environmental microbiology have demonstrated that water harbors a diverse array of microorganisms, with species and strains varying by location (Zhang et al., 2022). Plankton, including bacteria, are sensitive to environmental factors such as temperature, light, flow rate, pH, salinity, and electrolytes (Wang et al., 2013; Benbow et al., 2015). Our data revealed distinct community structures in different areas of the Pearl River, aligning with previous studies (Cannon et al., 2017; Zhao et al., 2018). As the distance from the estuary to the South China Sea decreased, the number of observed OTUs in water samples gradually decreased. Furthermore, we observed a higher abundance of certain salt-tolerant bacteria, such as *Rhodobacteraceae* and *Comamonadaceae,* in a specific location (W4), possibly due to seawater infiltration (Guo et al., 2020; Hu et al., 2020; Nishiwaki-Akine et al., 2022). When considering Bray–Curtis distance as a weighting factor, the differences within the same water sampling location were significantly higher compared to Jaccard distance, indicating that while the species composition in the same location remained relatively stable, their proportions dynamically changed due to water flow, resulting in increased intragroup differences under weighted conditions.

In this study, our objective was to analyze the bacterial composition of water samples collected from different locations in the Pearl River, as well as the corresponding drowned corpses. Our goal was to develop a novel bioinformatics method for diagnosing drowning sites. While it is expected that microorganisms found in the lung tissue of drowned individuals should theoretically have a strong correlation with those in the drowning medium, the variety of microorganisms in water and the fluidity of water can cause fluctuations in their relative abundance. Moreover, the lung tissue itself harbors a significant number of microorganisms, making it challenging to establish a direct link between the microorganisms in the tissue and the specific water source based solely on their types and abundance in water samples. To overcome these challenges, we employed a random forest modeling approach to identify important biomarkers at the genus level and their relative abundance in lung tissues of drowned rabbits from different locations. Through this approach, our model achieved a remarkable accuracy of 100% in inferring the drowning site. Among the top 20 bacterial genus taxa that had the most significant contribution to the random forest model, a majority of them (18/20) belonged to the phyla Firmicutes and Proteobacteria (Figure 5). Firmicutes are frequently observed to be highly abundant in decomposition studies, particularly in cadavers with longer postmortem intervals (Hyde et al., 2013; Procopio et al., 2019; Dash and Das, 2020). They are commonly found during the decomposition process. Proteobacteria are a group of bacteria that can function in both anaerobic and aerobic conditions (Liu et al., 2023), are known to be involved in the decomposition of organic matter and are reported as one of the most abundant taxa in decomposing cadavers (Hyde et al., 2013; Roy et al., 2021; Xiang et al., 2023). *Romboutsia*, a genus within the class Clostridia and family Peptostreptococcaceae, is commonly found in decomposition studies (Xiang et al., 2023). Clostridia, to which Romboutsia belongs, are considered ubiquitous in both the early and late stages of decomposition (Javan et al., 2017; Ohashi and Fujisawa, 2019) and have a significant impact on the accuracy of random forest models. The findings of this study highlight the importance of specific microbial taxa abundance in the lungs of drowned corpses and their association with the drowning site. Understanding this association is crucial for the further development and validation of microbiome methods used in forensic investigations for inferring drowning sites.

We attempted to utilize endemic species in water to infer the drowning site. However, we encountered challenges, as the endemic species identified in one site were also present in the lung tissues of drowned rabbits from other sites. Similar inconsistencies have been observed in our previous research regarding the consistency of diatoms in victim organs and drowning media (Li et al., 2022). We have identified four potential reasons for this inconsistency: (1) Water mobility can influence the species and abundance of microorganisms in both water samples and lung tissue of drowned rabbits. (2) The small volume (10–50 mL) of drowning medium may not fully represent the microbial composition of the water at the actual drowning site. (3) The volume of rabbit lung is relatively large, and the sampling procedure may introduce bias as only a small portion is used for testing. (4) The accuracy of species prediction at the species level using Qiime2’s plug-in q2-feature-classifier is low, which hampers specific species identification (Bokulich et al., 2018).

Our data indicated that greater differences among water samples provide more useful information for inferring the drowning site, but this method still has limitations. The relative abundance of microorganisms in blood and internal organs changes dynamically in different postmortem intervals (Can et al., 2014; Javan et al., 2016), which can potentially affect the accuracy of inferring drowning sites and requires further evaluation. Furthermore, it is important to note that our current research was primary research conducted on animals, which have a similar microbiome due to their consistent biological background. This similarity facilitates the discovery of differences caused by different processing factors. However, the situation is different in forensic practice, as humans have a more complex biological background and lifestyle (D’Argenio et al., 2021). Therefore, the information obtained should be further validated in humans. Notably, victims found in flowing rivers are usually far from drowning sites in many forensic practices (Zhang et al., 2020), and it is necessary to evaluate whether the differences in aquatic bacteria during the flow process affect the experimental results.

In conclusion, our data suggest that the random forest model based on the species and abundance of bacteria in the lung tissue of drowned individuals holds promise as a method for inferring the drowning site. However, the success of this method relies on the establishment of a comprehensive database of bacterial species and abundance in the lung tissues of drowned individuals across different water sources, which requires extensive systematic work. Future studies could consider implementing more rigorous protocols, including larger sample sizes, appropriate controls, and standardized procedures, to enhance the reliability of the results. Therefore, we sincerely hope that more forensic experts will participate in this work.

## Data availability statement

The datasets presented in this study can be found in online repositories. The names of the repository/repositories and accession number(s) can be found in the NCBI database at this link: https://www.ncbi.nlm.nih.gov/bioproject/PRJNA962514/.

## Ethics statement

The animal study was reviewed and approved by the Animal Ethics Committee of Southern Medical University.

## Author contributions

QS: conceptualization and writing – original draft. CY: investigation. LC: validation. YS: data analysis. QX: writing – review and editing. JZ: formal analysis. CL: design of the study and funding acquisition. HS: conceptualization and supervision. All authors contributed to the article and approved the submitted version.

## Funding

This work was supported by the Science and Technology Program of Guangzhou, China (Nos. 2019030012 and 202102080308) and Grant-in Aids for Scientific Research from Ministry of Public Security of the People’s Republic of China (2020GABJC38 and 2022JC35).

## Conflict of interest

The authors declare that the research was conducted in the absence of any commercial or financial relationships that could be construed as a potential conflict of interest.

## Publisher’s note

All claims expressed in this article are solely those of the authors and do not necessarily represent those of their affiliated organizations, or those of the publisher, the editors and the reviewers. Any product that may be evaluated in this article, or claim that may be made by its manufacturer, is not guaranteed or endorsed by the publisher.

## Footnotes

^1^ https://github.com/OpenGene/fastp

^2^ http://www.cbcb.umd.edu/software/flash
