# Supplementary material for: Inference of drowning sites using bacterial composition and random forest algorithm
Source: Front Microbiol. 2023 Jun 27;14:1213271. doi: 10.3389/fmicb.2023.1213271 (PMC10335767; doi:10.3389/fmicb.2023.1213271)
Supplement: Supplementary file 1 [file Data_Sheet_1.docx]

Supplementary Material


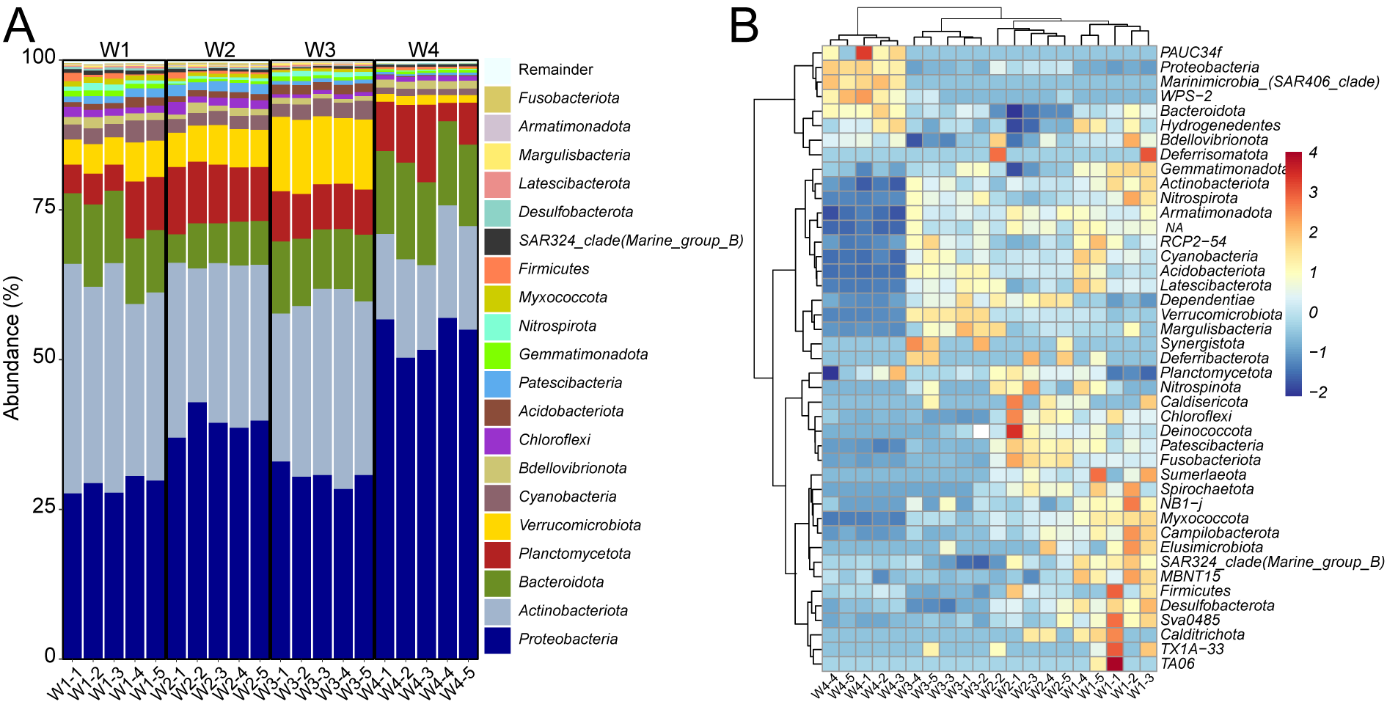


**Supplementary Figure 1.** Bacterial taxonomy of water samples at the Phylum level. (A) Bar plot. (B) Heatmap.


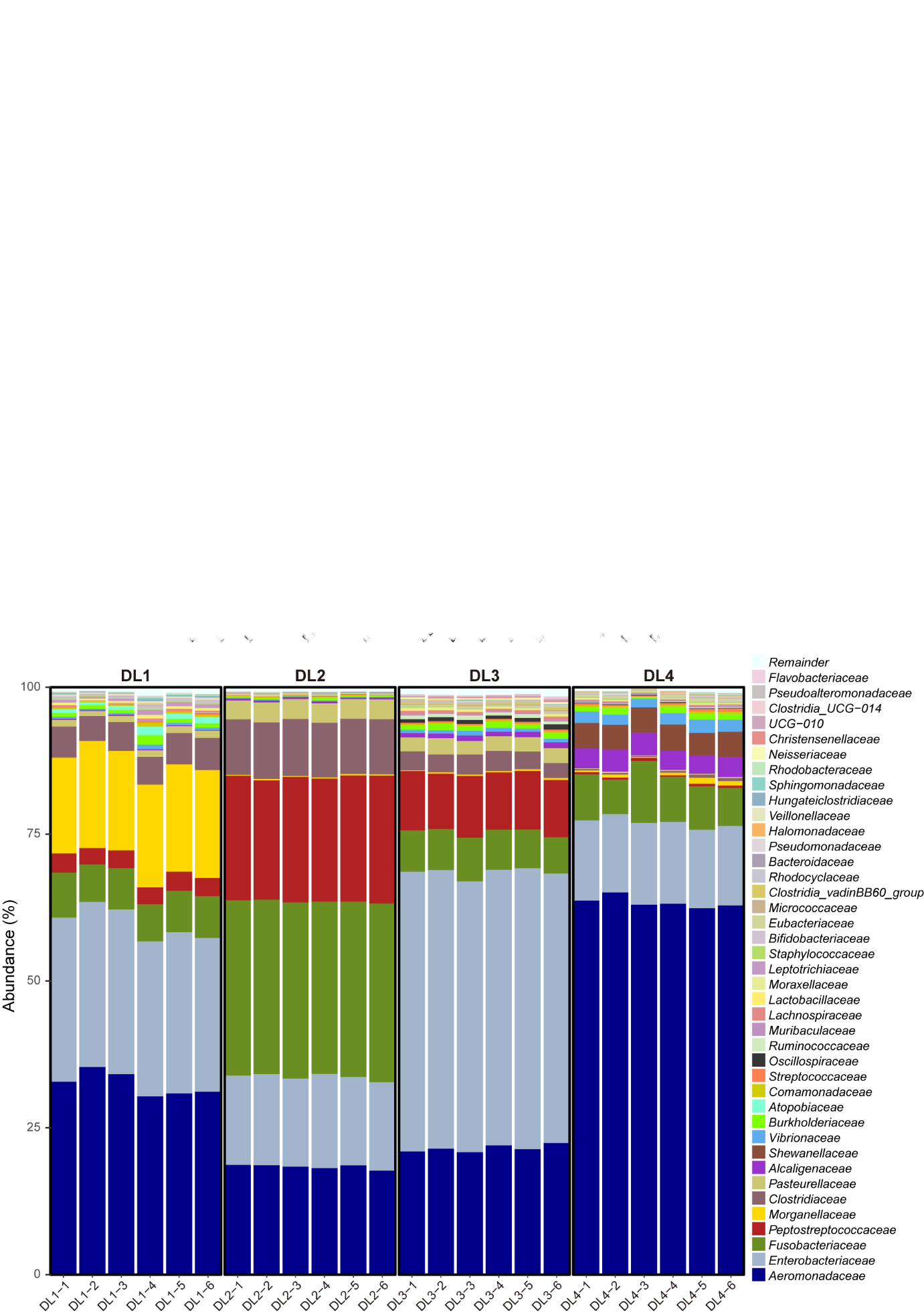


**Supplementary Figure** 2 Bacterial taxonomy of drown samples at the family level.


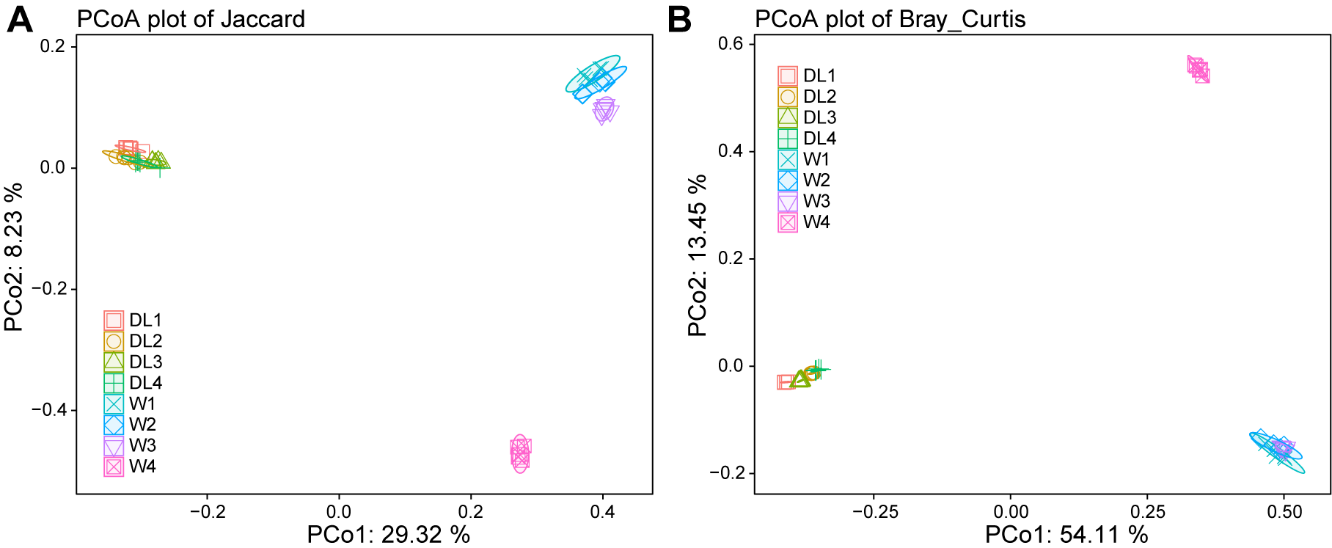


**Supplementary Figure** 3 Use PCoA of Jaccard (A) and Bray_curtis (B) distance to identify the relationship between drowned samples and water samples.


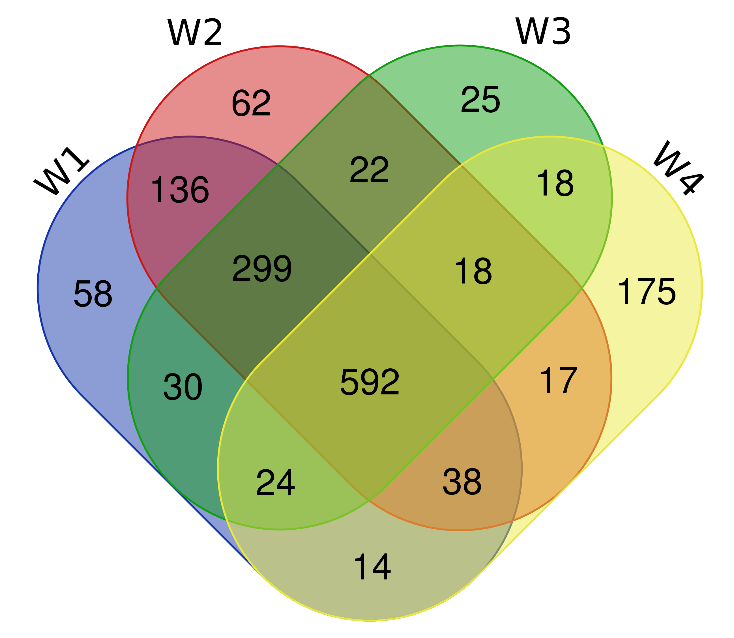


**Supplementary Figure** 4 Veen analysis of water samples at the species level.
